# Prevalence of Ochratoxin A in Human Milk in the Khorrambid Town, Fars Province, South of Iran

**DOI:** 10.5812/jjm.11220

**Published:** 2014-07-01

**Authors:** Parvin Dehghan, Keyvan Pakshir, Hossein Rafiei, Mostafa Chadeganipour, Mojtaba Akbari

**Affiliations:** 1 Department of Parasitology and Mycology, School of Medicine, Isfahan University of Medical Sciences, Isfahan, IR Iran; 2 Department of Medical Mycology and Parasitology, Basic sciences in Infectious Diseases Research Center, School of Medicine, Shiraz University of Medical Sciences, Shiraz, IR Iran; 3 Deputy of Research, School of Medicine, Isfahan University of Medical Sciences, Isfahan, IR Iran

**Keywords:** Mycotoxins, Ochratoxin A, Milk, Human

## Abstract

**Background:**

Ochratoxins belong to a group of mycotoxins produced as the secondary metabolites by filamentous fungi, such as *Aspergillus* and *Penicillium*. These toxins may be teratogenic, mutagenic, hepatotoxic, nephrotoxic, and may have immunosuppressive effects and pose a serious health problems to exposed humans and animals.

**Objectives:**

The present study aimed to determine the level of ochratoxin A (OTA) in the samples of mothers' milk in the Khorrambid Town, Fars Province, south of Iran.

**Materials and Methods:**

Between June and July 2011, samples of human milk were obtained from 87 mothers. The samples were diluted by absolute methanol at 1:4 ratio and after centrifugation, the supernatant was directly used to determine the level of OTA using competitive enzyme-linked immunosorbent assay (ELISA).

**Results:**

Among 87 human milk samples, 84 (96.6%) samples had positive results for OTA at a mean level of 24.57 ± 13.6 ng/L. According to the European Union Standard, 14 (16%) positive samples revealed more than the maximum limit of 40 ng/L for ochratoxin (range, 1.6-60 ng/L).

**Conclusions:**

Presence of OTA in the milk of mothers denotes a probable consumption of a contaminated foods. Therefore, regular monitoring of foods for presence of mycotoxins for lactating mothers seems necessary.

## 1. Background

Breastfeeding is highly important for the infants’ growth and health as well as their protection against many diseases. Based on the great advantages of breastfeeding, the World Health Organization (WHO) suggested that the infants should solely be fed by breast milk during the first six months of life (1). Although breast milk feeding has beneficial nutritional and immunological effects, there is the risk of mycotoxins entrance through consumption of the contaminated foods with molds and their metabolites (2). Mycotoxins are toxic secondary metabolites which are produced by filamentous fungi in suitable environmental conditions; thus, fungal growth in feeds should be avoided (3, 4).

Ochratoxins are a group of mycotoxins produced by mold fungi particularly *Penicillium verrucosum, P. nordicum, Aspergillus ochraceus, A. sulphureus, and A. niger *during the storage of cereals and other plant-derived products under non-optimal conditions (5). These fungi can contaminate several kinds of crops, vegetables, and fruits, which are used for human and animal consumption; therefore, their metabolites may be present in all kinds of raw and processed agricultural materials (3, 6, 7). Due to these toxic properties, regulations to control mycotoxins such as ochratoxins have been established in many countries (3, 7, 8).

In the recent few years there are increasing reports concerning food commodities such as grains, cereals, dried fruits, and milk contamination with ochratoxin A (OTA) (3, 9-13). OTA was shown to have carcinogenic and nephrotoxic effects. It has negative effects on the immune system function leading to teratogenesis and poses damages to DNA. Moreover, this toxin has also been reported to be related to Balkan (endemic) Nephropathy in humans (14, 15), and it has been considered as a contaminant of breast milk, as well (16). International Agency for Research on Cancer (IARC) has classified OTA in 2B category as a probable reason for human cancer (17).

In comparison to the adults, infants are more sensitive to the effects of mycotoxins because they have a lower weight but a higher metabolic rate. In addition, due to the incomplete growth of some of their organs and tissues, particularly the central nervous system, they have lower capability for neutralizing the toxins entering the body (6, 18). Best on our knowledge and after a comprehensive review of the literature, there was no study to determine the level of OTA in milk samples of Iranian mothers. Therefore, the present study was undertaken to determine the level of OTA in breast milk samples in Southern Iran.

## 2. Objectives

The natural prevalence of OTA in human milk from Iran had not been studied; hence, we aimed to determine the level of OTA in the mothers' milk samples in the town of Khorrambid, Fars Province, south of Iran.

## 3. Materials and Methods

In the current study, 87 breast milk samples were collected from seven health centers of Khorrambid Town from June to July 2011. They were evaluated for presence of OTA. The inclusion criteria were being a healthy breast feeder and having an infant younger than one year old. After making the necessary arrangements, the mothers were summoned to the health centers in the morning and were informed about the research objectives. A written informed consents was signed by participants and 5 to 10 mL of breast milk sample was collected in the sterile bottles. The samples were kept at -20ºC until measurement processes. Before starting the experiments, the samples were thawed. OTA level was determined in breast milks using a competitive enzyme-linked immunosorbent assay (ELISA) method (Helica Company, Santa Ana, California, USA).

According to the kit procedure, the breast milk samples were diluted by absolute methanol at 1:4 ratio and kept at room temperature for five minutes. They were centrifuged at ×3500 g and 10ºC for 15 minutes. The supernatant was used to determine the concentration of OTA. The required number of Microtiter wells was located in the plate, the standard solutions and the breast milk samples were added to the wells, and washed after each incubation session. All steps were performed according to the manufacturer’s instruction. The optical absorption of the samples was measured through the photometric method using ELISA reader at the wavelength of 450 nm. Afterwards, the calibration curve was drawn and used in order to determine the OTA concentration considering the samples’ absorption rate. Data were analyzed using SPSS version Version 16 (SPSS Inc., Chicago, IL, USA) employing Mann-Whitney U test, Kruskal-Wallis test, and correlation coefficient.

## 4. Results

The demographic data of the 87 mothers and their infants are presented in Table 1. Calculation of body mass index (BMI) showed that 11.5%, 28.7%, and 5.7% of the mothers under study were underweight, overweight, and obese, respectively, while 54% had normal BMI. Moreover, 55 (63.2%) and 52 (59.8%) mothers were involved in farming and ranching, respectively. Twenty subjects (23%) had miscarriage. Overall, 57 (65.5%), 24 (27.6%), and 6 (6.9%) mothers had under diploma, diploma, and above diploma education levels, respectively. Among the 87 breast milk samples, 84 mothers had mean OTA of 24.57 ± 13.6 ng/L (range, 1.6-60 ng/L).

**Table 1.** Anthropometric Characteristics of the Mothers and Their Infants and Their Association With the OTA Concentration in Breast Milk Samples ^a^

Variable	Mean ± SD	Ochratoxin A	
		r^b^	P Value
**Mother’s Age, y**	27.4 ± 5.18	0.13	> 0.05
**Mother’s Weight, Kg**	59.4 ± 10.34	0.02	> 0.05
**Mother’s Height, cm**	158.9 ± 5.4	-0.03	> 0.05
**Mother’s BMI**	23.5 ± 4.04	0.02	> 0.05
**Infant’s Age, mo**	5.26 ± 2.78	-0.02	> 0.05
**Infants’ Weight at Birth, Kg**	3.17 ± 0.52	-0.02	> 0.05
**Infants’ Height at Birth, cm**	49.21 ± 3.46	-0.04	0.07
**Infants’ Head Circumference at Birth, cm**	34.73 ± 1.66	-0.25	> 0.05

^a^ Abbreviation: BMI, body mass index.

^b^ Spearman Correlation Coefficient.

The results of Mann-Whitney U nonparametric test showed no significant association between OTA concentration and ranching (P > 0.05). Regarding OTA concentration, no significant difference was observed between the mothers who were involved in farming and those who were not (P > 0.05). Moreover, no significant association was noticed between the OTA concentration and miscarriage (P > 0.05). The results of Kruskal-Wallis nonparametric test revealed a statistically significant association between the mothers’ level of education and OTA concentration (P = 0.05). Furthermore, The OTA concentration of mothers’ milk in the villages where the sampling was done, was different from each other and it showed that OTA concentration was significantly associated with the residing villages of mothers (P = 0.039).

## 5. Discussion

In suitable conditions, various fungi such as *Aspergillus*, *Penicillium*, and *Fusarium* can easily contaminate the agricultural products during planting, growing, and harvesting (5). By consuming the foodstuff contaminated with the fungi producing such toxins, humans and animals are exposed to contamination with these mycotoxins (5, 19). Humans are contaminated with mycotoxins, such as OTA through different routes. Measurement of these toxins in the biological fluids of the body is a valuable index for examining the actual contamination with such toxins (20).

Since breast milk is a unique source for the infants’ nutrition and healthy growth, breastfeeding has been emphasized and encouraged all over the world. Therefore, mothers may face different natural or artificial contaminants and foodstuffs contaminated with various amounts of toxins during breastfeeding. Hence, consumption of healthy natural foodstuffs is of great importance for humans, particularly breastfeeding mothers (21).

Various reports from different countries revealed that human milk may contain OTA with different concentrations. In the present study, 84 (96.6%) out of the 87 breast milk samples were contaminated with OTA. The result of a similar study in Egypt indicated that from a total 120 human milk samples, OTA was found in 43 (35.8%) of the samples (22). In addition, the mean concentration of the toxin in this study was higher than that of the previous studies, such as 17.5 ng/L in Brazil (12), 10-57 ng/L in Italy (23,24), and 17-30 ng/L in Germany (16), while lower amounts such 8.87 ng/L was noticed in Egypt (25), 106 ng/L in Chile (26), 39.8 ng/L in Norway (27), and 7900 ng/L in Sierra Leone (28).

Moreover, OTA concentration in our study ranged from 1.6 to 60 ng/L, which is in agreement with the study performed in Slovakia (range, 2.3-60.3 ng/L). Nevertheless, the contamination rate was 96.6% in the current study, while it was reported as 30.2% in the Slovakian study (9). Up to now, a large number of investigations have been performed on the risks of consuming OTA and different values of tolerable daily intake (TDI) have been suggested (11, 18, 29). For instance, Nordic Working Group has suggested the highest daily TDI of OTA level in human's body as 5 ng/kg of baby weight (30). Recently, based on the regulations made by the European Union's cooperating with European Food Safety Authority (EFSA), the tolerable weekly intake of OTA has been reported as 120 ng/kg body weight and TDI of 20 ng has been suggested for the children (23).

In the current study, 14 (16%) out of the 84 (96.6%) positive samples showed more than the maximum limit of 40 ng/L of OTA. Assuming that an infant is 4 kg and consumes 500 mL milk every day, no more than 40 ng/L OTA must be present in the milk; however, the results of this study showed that 16% of the infants were exposed to the milk contaminated by more than permitted TDI of OTA. Although the short-term side effects of OTA have not been well identified in humans, the continuous absorption of this mycotoxin in the body and its long half-life in the blood (35 days) lead to accumulation of a large amount of this toxin in the infant’s body, which results in adverse effects over time (31). Recent studies on detecting of OTA in beans and dried fruits in Iran indicate that from 30 bean samples, three samples contained this toxin with the mean value of 0.29 ng/g; in addition, OTA was detected in 3.33% and 20% of dried apricots and prunes, respectively (10, 13). 

It seems that the environmental factors, such as temperature and humidity as well as the way that the foodstuffs and agricultural products are kept in this region, are the main reasons for high concentration of OTA in the mothers' milk. In fact, most of the people in the studied area have traditional lifestyles and they produce and preserve most of their necessary foodstuffs and agricultural products for a long time. The study results confirmed the existence of OTA in the breast milk of the mothers and consumption of the foodstuffs contaminated with such toxins. These toxins have long-term side effects; however, mothers are not recommended to stop breastfeeding their infants, as the advantages of breastfeeding are clear. 

In conclusion, comprehensive programs should be developed in order to regularly investigate and control these toxins in both humans’ and animals’ food chains so that the amount of these toxins can be reduced and their side effects can be prevented. Moreover, further studies in other parts of the country are recommended in order to identify the status of the society members’ exposure to these toxins. Monitoring of foods for the presence of mycotoxins like OTA and disposal of contaminated products should decrease the risk to the human and animal health. Therefore, regular monitoring of foods for presence of mycotoxins for lactating mothers seems necessary.
